# The Ubiquinone-Ubiquinol Redox Cycle and Its Clinical Consequences: An Overview

**DOI:** 10.3390/ijms25126765

**Published:** 2024-06-20

**Authors:** David Mantle, Mollie Dewsbury, Iain P. Hargreaves

**Affiliations:** 1 Pharma Nord (UK) Ltd., Morpeth NE61 2DB, UK; 2 School of Pharmacy and Biomolecular Sciences, Liverpool John Moores University, Liverpool L3 3AF, UK; m.r.dewsbury@2024.ljmu.ac.uk (M.D.); i.hargreaves@ucl.ac.uk (I.P.H.)

**Keywords:** coenzyme Q10, ubiquinone, ubiquinol, Q cycle, oxidoreductases, selenium

## Abstract

Coenzyme Q10 (CoQ10) plays a key role in many aspects of cellular metabolism. For CoQ10 to function normally, continual interconversion between its oxidised (ubiquinone) and reduced (ubiquinol) forms is required. Given the central importance of this ubiquinone–ubiquinol redox cycle, this article reviews what is currently known about this process and the implications for clinical practice. In mitochondria, ubiquinone is reduced to ubiquinol by Complex I or II, Complex III (the Q cycle) re-oxidises ubiquinol to ubiquinone, and extra-mitochondrial oxidoreductase enzymes participate in the ubiquinone–ubiquinol redox cycle. In clinical terms, the outcome of deficiencies in various components associated with the ubiquinone–ubiquinol redox cycle is reviewed, with a particular focus on the potential clinical benefits of CoQ10 and selenium co-supplementation.

## 1. Introduction

Coenzyme Q10 (CoQ10) is a member of a group of molecules known as ubiquinones, which are widely distributed in animals, plants, and micro-organisms. It consists of a central benzoquinone nucleus to which is attached a polyisoprenoid side chain comprising ten isoprene units. CoQ10 is one of the most lipophilic naturally occurring substances, located within intracellular membranes, principally in mitochondria. It is also located within the membranes of other subcellular organelles such as lysosomes, peroxisomes, and the Golgi apparatus. The benzoquinone ring of CoQ10 contains redox active sites, whereas the polyisoprenoid chain is responsible for positioning the molecule within the mid-plane of the lipid bilayer of various cell membranes (Crane, 2001) [1].

CoQ10 plays a key role in generating adenosine triphosphate (ATP) via oxidative phosphorylation within the mitochondrial electron transport chain (ETC). In addition, it also serves as an important lipid-soluble antioxidant and participates in the metabolism of fatty acids, amino acids, sulphides, and cholesterol. CoQ10 directly affects the expression of several genes, including those involved in inflammation. CoQ10 exists in both oxidised (ubiquinone) and reduced (ubiquinol) form. For CoQ10 to function normally in the above intracellular processes, continual interconversion between these two redox forms is required (Hargreaves et al., 2020) [2]. Due to the central importance of this ubiquinone–ubiquinol redox cycle, this article reviews the current information available about this process and its implications for clinical practice.

## 2. The Ubiquinone–Ubiquinol Redox Cycle

**Figure 1:**
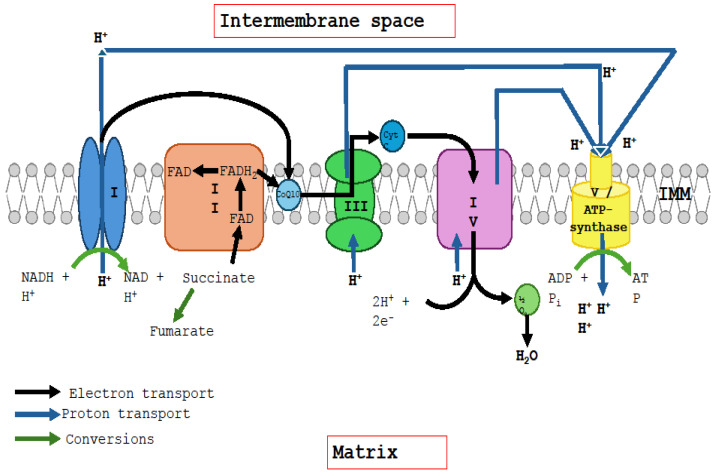
Mitochondrial electron transfer chain showing the enzymes and electron carriers involved in oxidative phosphorylation, the electron donators and direction of electron transport in the chain. CoQ10: coenzyme Q10, Cyt: cytochrome *c*, IMM: Inner mitochondrial membrane, I: complex I, II: complex II, III: complex III and IV: complex IV.

The ubiquinone–ubiquinol redox cycle comprises two steps. In step 1, ubiquinone is reduced to ubiquinol in association with Complexes I and II of the ETC; in step 2, ubiquinone is regenerated in association with Complex III. Reduced nicotinamide adenosine dinucleotide (NADH) acts as an electron donor for Complex I; succinate donates electrons to the flavin adenosine dinucleotide (FAD) prosthetic group of Complex II, reducing it to FADH2. The ETC is summarised in Figure 1, which the authors compiled based on data described in the present article.

### 2.1. Interaction of Ubiquinone with Complexes I and II

As noted above, CoQ10 exists in two principal forms, ubiquinone (oxidised form) and ubiquinol (reduced form), together with an intermediate ephemeral semi-quinone form. The fully oxidised ubiquinone form can be reduced by a two-step gain of two electrons and two protons to form the fully reduced ubiquinol form, passing through the semiquinone radical form. CoQ10’s ability to exchange two electrons as it converts between these forms helps it carry out various cellular functions, including energy production and antioxidant action. Each CoQ10 molecule undergoes approximately 5000 such redox cycles per hour, which is based on the requirement of two redox cycles to produce one molecule of ATP and that the body produces 70 Kg ATP per day, containing an estimated 2 g of CoQ10 (equivalent to 1.4 × 10^21^ CoQ10 molecules).

Within mitochondria, ubiquinone’s reduction to ubiquinol occurs principally during the electron flux from Complex I (NADH-ubiquinone oxidoreductase) and Complex II (succinate dehydrogenase) to Complex III (Q-cytochrome *c* oxidoreductase) in the electron transport chain. Complex I is a large protein comprising 45 subunits, located (as with Complexes II, III, and IV) within the inner mitochondrial membrane. Complex I receives two electrons from NADH (generated in the Krebs cycle and the beta-oxidation pathway) via a flavin mononucleotide (FMN) and transfers them along a chain of seven iron–sulphur centres to the reduction site where CoQ10 is reduced to ubiquinol. At the same time, this reaction is coupled with the pumping of four protons across the inner mitochondrial membrane and into the intermembrane space; thus, Complex I is a major contributor to the proton motive force driving ATP synthesis (Wikstrom et al., 2023) [3]. Complex II catalyses the oxidation of succinate to fumarate in the Krebs cycle, with the concomitant transfer of electrons from succinate to ubiquinone to form ubiquinol. Unlike Complex I (and Complexes III and IV), Complex II is not a proton pump and will not move protons into the intermembrane space because the oxidation of FADH2 to FAD at Complex II releases less energy than the oxidation of NADH to NAD+ at Complex I (Sousa et al., 2018) [4]. By gaining so many free electrons, Complexes I, II, and III are exposed to oxidative stress and may degrade if they cannot unload the accumulated electrons onto ubiquinone.

In addition to the interaction with ETC Complexes I and II, as outlined above, ubiquinone acts as an electron acceptor for several other mitochondrial inner membrane dehydrogenases, including choline dehydrogenase, dihydroorotate dehydrogenase, flavoprotein dehydrogenase, proline dehydrogenases 1 and 2, mitochondrial glycerol-3-phosphate dehydrogenase, and sulphide:quinone oxidoreductase, which are associated with metabolic pathways involving pyrimidine synthesis, fatty acid oxidation, amino acid metabolism, methylation, and hydrogen sulphide detoxification. As a result, while a deficiency of sulphide:quinone oxidoreductase can adversely affect the ubiquinone–ubiquinol interconversion process, deficient CoQ10 levels can interfere with the action of this enzyme, with concomitant effects on intracellular sulphide metabolism (Luna-Sanchez et al., 2017) [5], demonstrating the complex metabolic interdependencies within the ubiqinone–ubiquinol redox cycle. Ubiquinol is oxidised into ubiquinone by the so-called Q cycle, a process that occurs in the Complex III component cytochrome *b* (see following section).

### 2.2. The Q Cycle

The Q cycle is a process that occurs within ETC Complex III. Complex III (Q-cytochrome c oxidoreductase, cytochrome bc1 complex) has a complex structure comprising eleven subunits, whose functions are not fully elucidated. There are three main catalytic subunits comprising three major groups, namely cytochrome c1 (containing a single haem group), cytochrome *b* (containing two different haem groups), and the so-called Rieske iron–sulphur protein (containing a 2Fe-2S centre). In simple terms, the Q cycle describes the process in which electrons are transferred from ubiquinol to cytochrome *c*, with the release of four protons into the mitochondrial intermembrane space, and concomitant oxidation of ubiquinol to ubiquinone. The process comprises two half cycles. In the first half cycle, a ubiquinol molecule attaches to Complex III, transferring two electrons to the latter. One of these electrons moves onto the Rieske centre, from which it transfers to cytochrome *c1*, then finally to cytochrome *c*. The other electron follows a different pathway, moving through the haem groups of cytochrome *b* and then to a ubiquinone molecule to form a partially reduced semiquinone radical ion. The two protons originally attached to ubiquinol are transferred into the intermembrane space. In the second half-cycle, a second ubiquinol molecule attaches to Complex III, with the two transferred electrons following the same pathways as above, with two protons moving into the intermembrane space. The electron that moves to the Rieske centre eventually reduces a second cytochrome *c* moiety. The second electron combines with the semiquinone radical ion to form ubiquinol. In summary, a single Q cycle results in the formation of two reduced cytochrome c molecules, one ubiquinone molecule, one ubiquinol molecule, and four protons pumped into the mitochondrial intermembrane space (Crofts, 2021) [6].

## 3. Extramitochondrial Ubiquinone–Ubiquinol Redox: Role of Oxidoreductase Enzymes

The importance of the ubiquinone–ubiquinol redox cycle in normal mitochondrial function is well established; however, its role in the functioning of other subcellular organelles has yet to be fully elucidated, one example being in the normal functioning of lysosomes. In order for CoQ10 to fulfil such extra-mitochondrial functions, a mechanism for the interconversion of ubiquinone and ubiquinol forms exists. Outside the mitochondria, extramitochondrial ubiquinone is reduced by a diverse group of oxidoreductase enzymes, the most efficient of which is the selenoenzyme thioredoxin reductase together with lipoamide dehydrogenase and glutathione reductase (Xia et al., 2003) [7]. All three of these enzymes, as members of the pyridine nucleotide disulfide oxidoreductase family, have a similar structure and mechanism of action. Thioredoxin reductase and glutathione reductase use nicotinamide adenine dinucleotide phosphate (NADPH) as a cofactor to transfer a pair of electrons, via FAD, to a conserved disulfide redox centre, while lipoamide dehydrogenase uses NADH instead of NADPH. Whilst these enzymes also exist within mitochondria, ubiquinone reduction within mitochondria occurs principally in association with ETC Complexes I and II. In general terms, thioredoxin reductase (TrxR1) is a selenium-containing cytosolic enzyme, which catalyses the transfer of electrons from NADPH to thioredoxin protein, which can then reduce a wide range of chemically unrelated compounds (Tinkov et al., 2018) [8]. TrxR2 is the mitochondrial equivalent of thioredoxin reductase, which acts as a major free radical scavenging system in mitochondria (Go et al., 2008; Huang et al., 2015) [9,10].

Although the principal target for the TrxRs enzyme is thioredoxin, the cytosolic form can regenerate several important antioxidants, including ubiquinol. The efficacy of TrxR-1 at the cellular level has been established using transfected TrxR-1 overexpressing stable cell lines. Homogenates and cytosols derived from the overexpressing cells were found to reduce ubiquinone more efficiently than control cell homogenates and cytosols. Furthermore, adding increased concentrations of selenium to the transfected cells increased TrxR-1 activity accompanied by increased ubiquinone reductase activity (Xia et al., 2003) [7]. In summary, these data suggest that mammalian cytosolic TrxR-1 is the most efficient ubiquinone reductase identified to date. They also indicate the close relationship between ubiquinone/ubiquinol and the essential trace element selenium.

Lipoamide dehydrogenase occurs principally within mitochondria as a component of the pyruvate dehydrogenase and 2-oxoglutarate dehydrogenase complexes as well as in cytoplasmic isoenzyme forms. Lipoamide dehydrogenase catalyses the reversible re-oxidation of dihydrolipoic acid and NADH:NAD(+) transhydrogenation and can transfer electrons from NADH to various redox-active compounds, including ubiquinone (Nordman et al., 2003) [11]. Glutathione reductase is an enzyme responsible for the maintenance of intracellular levels of antioxidant-reduced glutathione by catalysing glutathione disulphide reduction in the presence of NADPH and FAD. It also reduces ubiquinone (Nordman et al., 2003) [11]. Glutathione reductase occurs in both mitochondrial and cytoplasmic isoenzymatic forms. Nordman et al. [11] found that ubiquinone reduction occurs at the active site of the enzyme. This finding suggests that glutathione reductase may have less capacity to reduce ubiquinone because of competition with oxidised glutathione reduction.

Other enzymes involved in the extramitochondrial cycling of ubiquinone/ubiquinol include DT-diaphorase (also referred to as NOQ1 or NAD(P)H: quinone acceptor oxidoreductase), a flavoprotein catalysing the two-electron reduction of quinones to hydroquinones, using either NADH or NADPH as the electron donor (Beyer et al., 1997) [12], and NADPH-dependent coenzyme Q reductase, a cytosolic flavoprotein that reduces ubiquinone by a two-electron reduction mechanism (Takahashi et al., 1996) [13]. However, the quinone reductase activity of DT-diaphorase decreases with the increasing number of isoprene units in the quinone side chain. This enzyme has relatively low reductase activity compared to human ubiquinone, which contains 10 isoprene units in its side chain (Lind et al., 1990) [14].

## 4. Clinical Implications of the Ubiquinone–Ubiquinol Redox Cycle

The clinical implications of the ubiquinone–ubiquinol redox cycle are divided into two sections: The first section discusses the clinical consequences of deficiencies in various components of the cycle, including ETC Complex I, ETC Complex II, thioredoxin reductase, lipoamide dehydrogenase, glutathione reductase, and DT-diaphorase, as well as CoQ10 itself. The second section discusses the clinical benefits of the supplementary combination of CoQ10 and selenium, particularly the KiSel-10 randomised controlled trial carried out in an elderly population.

### 4.1. Deficiencies of Redox Cycle Components: Complexes I, II, and III

**ETC Complex I deficiency** typically manifests in early childhood, with death often occurring in the first year of life. Patients may present a wide range of symptoms, including lactic acidosis, neurological problems (intellectual disability, ataxia, dystonia, epilepsy, or encephalopathy), liver or renal disease, and cardiomyopathy. Complex I deficiencies may result from mutations in mitochondrial or nuclear genes. Of the 45 protein subunits comprising Complex I, seven are encoded by mitochondrial DNA and 38 are nuclear-encoded (Swalwell et al., 2011) [15]. There is also the possibility of mutations in genes encoding at least 19 proteins, such as acyl-coA dehydrogenase 9, involved in the correct assembly of various subunits comprising Complex I (Repp et al., 2018) [16]. Treatment of Complex I deficiency based on individual or small groups of patients has focused principally on supplementation with riboflavin (as a component of the flavin mononucleotide prosthetic group of Complex I) as well as supplementation with L-carnitine (as a chaperone of long-chain fatty acids in mitochondria) (Bernsen et al., 1991; Bernsen et al., 1993; Scholte et al., 1995; Ogle et al., 1997; Artuch et al., 2006; Curtabbi et al., 2024) [17,18,19,20,21,22].

**Complex II deficiency** is an autosomal recessive multisystemic metabolic disorder with a highly variable phenotype. Onset is typically in infancy, with multisystem involvement of the brain, cardiac, and skeletal muscles. Complex II is the smallest complex in the mitochondrial electron transport system, comprising only four subunits. Unlike the other complexes in the mitochondrial electron transport chain, Complex II is encoded entirely by autosomal genes. To date, 61 patients with Complex II deficiency have been described, associated with 32 different pathogenic variants in four distinct Complex II genes: three structural subunit genes (*SDHA*, *SDHB*, and *SDHD*) and one assembly factor gene (*SDHAF1*). Some cases of Complex II deficiency have reported clinical improvement following supplementation with riboflavin. Similar to Complex I above, riboflavin is a component of Complex II’s FAD cofactor. Clinical improvement of Complex II deficiency has also been reported following supplementation with L-carnitine and CoQ10 (Fullerton et al., 2020) [23].

As noted above, in addition to its role in electron transport and proton translocation, the ubiquinone–ubiquinol redox cycle depends on Complex III’s actions in re-oxidising ubiquinol to ubiquinone. **Complex III deficiency** results from mutations in genes encoding various subunits of Complex III or its assembly factors. The most common cause of Complex III deficiency is mutations in the gene coding for the BCS1L chaperone protein, which has only one transmembrane domain and belongs to the AAA ATPase family. More than 30 different mutations in the gene encoding BCS1L have been identified, with the resulting phenotypes varying from the lethal neonatal GRACILE (growth restriction, aminoaciduria, cholestasis, iron overload, lactic acidosis, early death) syndrome, to a comparatively mild form of Bjornstad syndrome characterised by neurosensory hearing loss but an otherwise normal lifespan (Fernandez-Vizarra and Zeviani, 2015; Banerjee et al., 2022) [24,25].

### 4.2. Extramitochondrial Oxidoreductase Enzyme Deficiency

Thioredoxin reductase catalyses hydrogen peroxide reduction as an intracellular defence against oxidative damage induced by reactive oxygen-free radical species. Regarding deficiencies in general terms, rats fed a selenium-deficient diet experienced a significant decrease in thioredoxin reductase activity in some tissues, particularly in the liver and kidney (4% and 11% of control, respectively) (Hill et al., 1997) [26]. For TrxR1, the cytoplasmic form of thioredoxin reductase, a study in knockout mice showed that reduced TrxR1 activity was associated with increased oxidative stress, leading to the development of heart disease (Yamamoto et al., 2003) [27]. Only one clinical study was relevant to TrxR1 deficiency. Pei et al. (2013) [28] implicated TrxR1 deficiency in the pathogenesis of Keshan disease (a type of cardiomyopathy associated with selenium deficiency) since myocardium samples from patients had reduced TrxR1 levels (and increased oxidative stress) compared to controls.

Research on lipoamide dehydrogenase deficiency has mainly focused on the mitochondrial form of the enzyme, which is involved in energy metabolism as part of the pyruvate dehydrogenase complex within the mitochondrial matrix (Szabo 2023) [29]. No clinical studies were identified relating to a deficiency in the cytoplasmic form of lipoamide dehydrogenase. Glutathione reductase deficiency is a rare disorder where glutathione reductase activity is absent from erythrocytes and/or leukocytes. Mutations in the gene encoding glutathione reductase or a nutritional deficiency of the enzymatic co-factor riboflavin can cause glutathione reductase deficiency (Couto, 2016) [30]. No publications were identified regarding glutathione deficiency and the ubiquinone–ubiquinol redox cycle.

### 4.3. CoQ10 Deficiency

CoQ10 deficiencies can be the result of primary or secondary deficiencies in CoQ10 biosynthesis. Primary CoQ10 deficiency is caused by mutations in genes involved in the CoQ10 biosynthetic pathway. CoQ10 biosynthesis is a complex multistep process that occurs in different subcellular locations. The polyisoprenoid tail of CoQ10 is synthesised (via polyprenyl diphosphate synthase) via the mevalonate pathway in the cytoplasm, which is attached to the benzoquinone ring (originating from tyrosine and its metabolite 4-hydroxybenzoate) within the mitochondrion. The benzoquinone nucleus is then subjected to further hydroxylation, methylation, and decarboxylation enzymatic modifications. To date, mutations in ten genes, *COQ1* to *COQ10* (which encode from the proteins COQ1 to COQ10, respectively) involved in the biosynthetic pathway have been identified, as outlined below:

COQ1 (heterotetrameric decaprenyl diphosphate synthase), consisting of PDSS1 and PDSS2, is involved in polyisoprenoid chain synthesis.

COQ2 (4-hydroxybenzoate polyprenyl transferase) is involved in the condensation of the isoprenoid chain with the benzoquinone ring.

COQ3, COQ5, COQ6, and COQ7 are involved in the methylation, decarboxylation, hydroxylation, and deamination of the benzoquinone nucleus.

COQ8A is necessary for the phosphorylation of COQ 3, 5, and 7.

The COQ9 lipid-binding protein is necessary for stabilising COQ7, and COQ10 appears to direct CoQ10 localisation within the mitochondrial membrane (Mantle et al., 2023) [31].

Primary CoQ10 deficiencies are associated with a heterogeneous spectrum of clinical presentations; however, the following generalisations can be made: (i) The disorder typically has a neonatal, infantile, or childhood age of onset; (ii) patients present with a predominantly neurological presentation including encephalopathy, psychomotor delay, cerebellar atrophy/ataxia, and optic atrophy. In addition, patients can also present with nephropathy, cardiomyopathy, or any combination thereof; (iii) the clinical outcome for patients typically ranges from severe disability to fatality. To date, around 300 patients with primary CoQ10 deficiency have been identified; however, many of these patients may not have been reported in the literature. In approximately 100 of the 300 patients who reported primary CoQ10 deficiency, CoQ10 supplementation was undertaken, as described in the present review. Some of the above syndromes can be remedied by oral CoQ10 supplementation, particularly when diagnosed in the early stages of the disease. However, the response to CoQ10 supplementation depends on particular mutated genes and the location of the mutation within each of the respective genes. The steroid-resistant nephrotic syndrome resulting from COQ mutations appears particularly amenable to CoQ10 supplementation (Mantle et al., 2023) [31].

Secondary coenzyme Q10 deficiency results from mutations in genes not directly involved in the CoQ10 biosynthetic pathway or from non-genetic factors associated with several different diseases and conditions. Contrary to primary CoQ10 deficiency, secondary CoQ10 disorders are relatively common. They can occur for a variety of reasons, including mutations in genes not directly related to the synthetic pathway, oxidative stress-induced CoQ10 reduction, and the effects of pharmacological agents such as statins. Examples of secondary CoQ10 deficiency resulting from genetic/non-genetic causes include mutations in the *APTX* gene encoding the protein aprataxin in ataxia oculomotor aprataxin 1 disorder, the *BRAF* gene encoding the enzyme serine/threonine-protein kinase B-Raf in cardiofaciocutaneous syndrome, multiple acyl-CoA dehydrogenase deficiencies (by mutations in the *ETFDH* gene), and spinocerebellar ataxia-10 (by mutations in the *AN010* gene). Depleted CoQ10 levels have been reported in several secondary deficiencies. Randomised controlled clinical trials have shown supplementation with CoQ10 to be of significant benefit, particularly in heart failure, chronic kidney disease, type II diabetes, reproductive disorders, and fibromyalgia (Mantle et al., 2022) [32].

### 4.4. Clinical Benefits of CoQ10 and Selenium Co-Supplementation

This section of the article relates to the synergistic interaction between supplementary CoQ10 and selenium and its clinical consequences, particularly in KiSel-10 randomised controlled clinical trials. In this study comprising 443 elderly Swedish subjects (aged 70–88), long-term (4 years) supplementation with CoQ10 (200 mg/day) and selenium (200 mcg/day) resulted in a 53% reduction in cardiovascular mortality risk. Echocardiography showed significantly better cardiac function in supplemented participants, who experienced a significant reduction in both hospital days and deterioration in health-related quality of life, compared to untreated patients (Alehagen et al., 2013; Johansson et al., 2015) [33,34]. There were significant reductions in the blood levels of pro-BNP (Johansson et al., 2013) [35], C-reactive protein, and soluble platelet selectin (sP selectin) as markers of inflammation (Alehagen et al., 2015a) [36], and copeptin and adrenomedullin as markers of oxidative stress (Alehagen et al., 2015b) [37]. A follow-up study reported combined CoQ10 and selenium supplementation’s ability to reduce long-term cardiovascular mortality risks; this effect persisted for eight years after the end of the intervention period (Alehagen et al., 2015c; Alehagen et al., 2018) [38,39].

CoQ10 and selenium have key roles in heart function and are likely to be deficient in the elderly population. Most of the body’s daily requirement of CoQ10 is provided by endogenous synthesis. In humans, the capacity for endogenous synthesis declines with age, such that blood CoQ10 levels in a 65-year-old may be approximately half for a 25-year-old. In addition, the level of CoQ10 in the heart muscles of 19–21-year-old individuals was 110 mcg/g compared to 77–81-year-old individuals with 47 mcg/g (Kalen et al., 1989) [40]. Dietary intake of selenium in many European countries, including Sweden and the UK, is reported to be suboptimal and more pronounced in the elderly population. Amongst the elderly Swedish population studied, those with the lowest levels of blood selenium were found to be at increased risk of cardiovascular mortality (Alehagen et al., 2016) [41]. Further details of the KiSel-10 study are summarised in Table 1 and Table 2, respectively. The age-related decline in endogenous CoQ10 synthesis, suboptimal dietary intake of selenium, and the associated increased risk of heart disease and cardiovascular mortality provided support for the treatment regime utilised in the KISEL-10 study. In mechanistic terms, CoQ10 supplementation alone is likely to be suboptimal if individuals are also deficient in selenium. In this regard, the role of selenium as a co-factor for the enzyme, thioredoxin reductase, and the importance of the latter in the ubiquinone–ubiquinol redox cycle (as noted in Section 3 of this article) is likely to be a key factor.

Other areas of clinical practice where the combination of supplementary CoQ10 and selenium may be of benefit include myalgic encephalomyelitis/chronic fatigue syndrome (ME/CFS) (Castro-Marrero et al., 2022) [49], reproductive medicine (Alahmar and Sengupta, 2021) [50], and critical care medicine (Hargreaves and Mantle, 2019) [51]. In patients with ME/CFS, co-supplementation with 400 mg CoQ10 and 200 mcg selenium per day for 8 weeks resulted in reduced levels of oxidative stress and inflammation biomarkers, together with improved fatigue severity and quality of life. In reproductive medicine, co-supplementation of infertile men with 200 mg CoQ10 and 200 mcg selenium daily for 3 months resulted in improved antioxidant status, sperm concentration, and motility. In critical care medicine, multiple organ dysfunction and resultant mortality in critically ill patients are associated with impaired cellular energy metabolism and oxidative stress. Clinical studies that have used selenium supplementation based on its role as a key cofactor of multiple antioxidant enzymes have reported variable outcomes in critically ill patients. However, the synergistic interaction between selenium and coenzyme Q10 has not been considered in such studies. As noted above, the selenoenzyme thioredoxin reductase has an important role in normal CoQ10 functioning. CoQ10 participates in the biosynthesis of selenocysteine, which is required for the synthesis of selenoproteins such as thioredoxin reductase (Moosmann and Beal, 2004) [52]. Thus, selenium deficiency can result in reduced CoQ10 recycling, and concomitant CoQ10 deficiency can result in reduced selenoprotein synthesis.

## 5. Summary

In this article, we review current information available on the mechanism of the ubiquinone–ubiquinol redox cycle, a process essential for normal CoQ10 functioning and, hence, normal cell functioning. Data presented in the present article were obtained by searching the Medline database of the National Library of Medicine, which in the authors’ view represents the most reliable database for searching peer-reviewed medical literature.

Whilst the mechanism by which ubiquinone is reduced to ubiquinol by ETC Complexes I or II (and ubiquinol re-oxidised to ubiquinone by Complex III) within mitochondria is now well established, the mechanism by which ubiquinone is reduced to ubiquinol outside of the mitochondria requires further research, particularly the role of extramitochondrial oxidoreductase enzymes in this process. It is predicted that clinical cases corresponding to novel genetic mutations resulting in ETC Complexes I and III (particularly Complex III) deficiencies will continue to be described. In addition, novel data relating to reduced ubiquinone continue to be presented, an example being the reduction in ubiquinone by the mitochondrial-associated selenoenzyme sulphide quinone oxidoreductase (SQOR) and its role in suppressing ferroptosis (Lee et al., 2024) [53]. This study demonstrated a previously unknown role for selenium, unrelated to its role as a co-factor in selenoproteins, where the selenium metabolite selenide can transfer electrons to ubiquinone to generate ubiquinol, catalysed by SQOR.

In clinical terms, the outcome of deficiencies in the various components of the ubiquinone- ubiquinol redox cycle, the role of selenium as a cofactor of the oxidoreductase enzyme thioredoxin reductase, the importance of the latter enzyme in the ubiquinone- ubiquinol cycle, the subsequent synergistic interaction between CoQ10 and selenium, and its application in the KiSel-10 clinical study have all been reviewed in the present article.

With regard to the deficiency of components directly involved in the redox cycle in more general terms, only two components (CoQ10 as ubiquinone/ubiquinol and NAD as NAD+/NADH) are commercially available in supplement form. Of these two substances, CoQ10 is by far the best characterised; the bioavailability is well established (Mantle & Dybring, 2020) [54], and the efficacy and safety of supplemental CoQ10 have been described in a considerable number of randomised controlled clinical trials (Mantle et al., 2022) [32]. The bioavailability of orally administered NAD+ or NADH is uncertain (She et al., 2021) [55], and intravenous infusion of NAD+ serves as an alternative method of administration by-passing the intestinal tract (Braidy et al., 2019) [56]. Although some randomised controlled trials supplementing NAD+ or NADH have been undertaken (Castro-Marrero et al., 2021) [57], many such studies have supplemented precursor forms of NAD such as β-nicotinamide mononucleotide (NMN) (Yi et al., 2023) [58].

Of the other main components of the redox cycle, Complexes I, II, and III are large protein complexes. They are not available in supplemental form and are unlikely to survive transit through the stomach or be subsequently absorbed from the intestinal tract. Although a small amount of dietary protein may survive transit through the stomach, this is unlikely to apply to large protein complexes (Dallas et al., 2017) [59]. Similarly, the absorption of large protein complexes from the intestinal tract is unlikely to occur (Gardner, 1998) [60]. However, in cases of redox cycle Complex deficiencies, there is evidence that compensatory mechanisms facilitate ATP production, supporting the latter via appropriate supplementation. Zielinski et al.’s computer modelling study [61] based on human cardiomyocyte mitochondrial metabolism predicted that Complex I deficiency could be compensated for via multiple pathways. Complex II deficiency had less metabolic flexibility because it impacted both the TCA cycle and the electron transport chain. Complex III and IV deficiencies caused the greatest decreases in ATP production, with metabolic consequences paralleling hypoxia. Compensatory mechanisms for Complex I deficiency include the glycerol-3-phosphate shuttle, the folate shuttle, and increased beta-oxidation of fatty acids. For example, in mammalian mitochondria, the glycerol-3-phosphate shuttle links glycolysis, oxidative phosphorylation, and fatty acid metabolism. In this shuttle, dihydroxyacetone phosphate is converted to glycerol-3-phosphate by a cytoplasmic glycerol-3-phosphate dehydrogenase 1 via oxidizing one molecule of NADH to NAD+. Glycerol-3-phosphate is then converted back to dihydroxyacetone phosphate by mitochondrial glycerol-3-phosphate dehydrogenase 2, which reduces one molecule of FAD to FADH2. FADH2 then enters mitochondrial respiration by reducing ubiquinone to ubiquinol, generating ATP (Mracek et al., 2013) [62]. Compensatory mechanisms identified for Complex II deficiency include the pentose phosphate pathway (TeSlaa et al., 2023) [63] and the folate shuttle (Zheng and Cantley, 2019) [64]. No compensatory mechanisms have been identified for Complex III or IV deficiencies.

In another example using a model based on Complex I-deficient patient-derived fibroblasts, Schirris et al. [65] predicted that increased cholesterol production, export, and utilization would counterbalance the surplus of reduced equivalents in patient-derived fibroblasts (since these pathways consume considerable amounts of NAD(P)H). They also predicted that fibrates attenuated increased NAD(P)H levels and improved Complex I-deficient fibroblast growth by stimulating cholesterol production via enhancement of its cellular efflux. In Complex I-deficient (Ndufs4^−/−^) mice, fibrate treatment resulted in prolonged survival and improved motor function, accompanied by increased cholesterol efflux from peritoneal macrophages. The effect of similar interventions on human patients with such deficiencies, which remains to be investigated, represents future areas of research.

## Figures and Tables

**Table 1:** Increase in serum CoQ10 and selenium concentrations in the KiSel-10 study’s combined supplementation of community-living elderly citizens after 48 months (from Alehagen et al., 2020 [42].

Supplement	Baseline Serum Level	End of Study Serum Level
Coenzyme Q10	0.82 mg/L	2.17 mg/L
Selenium-enriched yeast	67.1 μg/L	210.3 μg/L

**Table 2:** Statistically significant outcomes in the KiSel-10 study’s combined CoQ10 and selenium supplementation of community-living elderly citizens.

Reference	Change in Outcome Variable after 48 Months of Combined Supplementation	Statistical Significance
Alehagen et al. [33]	Reduced cardiovascular mortality in the active treatment group vs. the placebo group (5.9% vs. 12.6%)	*p* = 0.015
Alehagen et al. [35]	Lower NT-proBNP level values in the active treatment group vs. the placebo group (mean values: 214 ng/L vs. 302 ng/L)	*p* = 0.014
Alehagen et al. [33]	Better cardiac function score in echocardiography for the active treatment group vs. the placebo group	*p* = 0.03
Alehagen et al. [36]	Improved CRP levels (from of 4.1 ng/mL down to 2.1 ng/mL) in the active treatment group vs. a slight rise (from 4.8 ng/mL up to 5.1 ng/mL) in the placebo group	*p* = 0.009
Alehagen et al. [37]	Less increase in copeptin and MR-proADM levels in the active treatment group vs. the placebo group	*p* = 0.031 *p* = 0.026
Johansson et al. [34]	More days out of hospital in the active treatment group vs. the placebo group (1779 compared with 1533)	*p* = 0.03
Johansson et al. [34]	Less decline in health-related quality of life domains, including physical performance, vitality, and overall quality of life, in the active treatment group vs. the placebo group	*p* = 0.001
Alehagen et al. [43]	Lower D-dimer concentrations in the active treatment group (0.22 mg/L) vs. the placebo group (0.34 mg/L)	*p* = 0.006
Opstad et al. [44]	Less shortening of leukocyte telomere length in the active treatment group (+0.019) vs. the placebo group after 42 months (−0.129)	*p* = 0.02
Opstad et al. [45]	Increased SIRT1 concentration in the active treatment group (from 252 to 469 ng/mL) vs. decreased SIRT1 concentration in the placebo group (from 269 down to 190 ng/mL)	*p* = 0.006
Dunning et al. [46]	Increased serum sulphydryl levels, reduced cardiovascular disease risk	*p* = 0.002
Alehagen et al. [47]	Reduced serum levels of fibroblast growth factor 23, reduced risk of cardiovascular disease	*p* = 0.01
Alehagen et al. [48]	Serum markers of renal dysfunction (creatinine, cystatin-C) reduced	*p* = 0.001
